# A stochastic differential equation model for transcriptional regulatory networks

**DOI:** 10.1186/1471-2105-8-S5-S4

**Published:** 2007-05-24

**Authors:** Adriana Climescu-Haulica, Michelle D Quirk

**Affiliations:** 1 Laboratoire Biologie, Informatique, Mathématiques, Institute de Recherche en Technologies et Sciences pour le Vivant CEA, 17 Rue des Martyrs, Grenoble, 38052 France; 2 Decision Applications Division, Los Alamos National Laboratory, MS K551, Los Alamos, New Mexico, 87545 USA

## Abstract

**Background:**

This work explores the quantitative characteristics of the local transcriptional regulatory network based on the availability of time dependent gene expression data sets.

The dynamics of the gene expression level are fitted via a stochastic differential equation model, yielding a set of specific regulators and their contribution.

**Results:**

We show that a beta sigmoid function that keeps track of temporal parameters is a novel prototype of a regulatory function, with the effect of improving the performance of the profile prediction. The stochastic differential equation model follows well the dynamic of the gene expression levels.

**Conclusion:**

When adapted to biological hypotheses and combined with a promoter analysis, the method proposed here leads to improved models of the transcriptional regulatory networks.

## Background

The production of independent sets of time courses of microarray data [1-3], obtained for the most studied eukaryotic organism *Saccharomyces cerevisiae*, improved the knowledge on the relationship between genes through the transcriptional process in the cell. The mechanism of the gene expression regulation is not entirely known, yet progress has been made by combining *in silico *approaches with the analysis of experimental data. In particular, contributions from a qualitative analysis realized by the recognition of specific promoter sequences, binding sites, and transcription factors are enhanced by quantitative studies obtained from microarray gene expression data [4,5]. The transcriptional regulatory network, built from thousands of genes, has a dynamical nature: the transcriptional program adapts itself to organismal development through the cell cycle, or as a response to changes in environment. In a systemic view the network architecture is potentially established by a qualitative analysis while quantitative methods address the main dynamical aspects – the network switches and the level of its parameters. This type of information may be obtained by processing gene expression data that keep track of the variations in the experimental conditions and temporal modifications suited for the understanding of a particular transcriptional behavior.

A mathematical model for the processing of time dependent gene expression data has been sought to describe the dynamical aspects of regulation and to estimate the level of contribution for each transcriptional regulator in a succession of events. In this work we strengthen the model proposed in [6], by means of a novel pattern for the regulatory function. This model uses a SDE to describe the dynamics of the target mRNA expression level that reflects the actual knowledge about the stochasticity in gene expression, in a biological framework [7]. The drift term of the SDE depends on the regulatory rate of the target gene. The noise term is modeled by a Brownian Motion process which accounts for the superposition of small random factors that arise dynamically. The regulation rate is obtained as a linear combination of the regulatory functions of specific elements of the network. We propose a beta sigmoid function as the prototype of the regulatory function, designed to keep track of the local temporal patterns of the target gene regulators.

Our analysis shows that the utilization of the beta sigmoid function enhances the results in [6] where sigmoid functions were considered. The comparison was made by applying the model to the same test data set as in [6], given by gene expression measurements of the mRNA levels of 6178 *S. cerevisiae *ORFs at 18 time points under the *α *factor synchronization method [1]. A candidate pool of potential regulators was constructed by joining transcription factors, cell-cycle control factors and DNA-binding transcriptional regulators as found in the literature [1,8]. We performed the same statistical analysis from [6] based on the maximum likelihood principle for the estimation of the model parameters. The AIC strategy was used for the selection of the best fitting combination of the pool regulators. With the addition of beta sigmoid pattern, the SDE model renders good prediction results even in the case of the previously worst fitted genes obtained by [6]. The procedure proposed herein may be well suited to quantify transcriptional regulatory networks, provided it is tailored to the characteristics of the input data set.

## Results

The model was evaluated on the data set from [1] that provides gene expression measurements of the mRNA levels of 6178 S. cerevisiae ORF s at 18 time points under the *α *factor synchronization method. To compare our results with those from [6], we used the data set of 216 potential regulator listed at [9], constructed by joining transcription factors, cell-cycle control factors and DNA-binding transcriptional regulators described in the literature [1,8,10]. This set has been created with respect to the regulation of the cell cycle process. There are about 800 genes identified to be involved in the cell cycle of the budding yeast [1] and we performed our analysis on the entire data set. This fact did not carry any methodological artifact because the target genes are processed independently. The advantage is that good prediction results may imply new hypothesis about the regulators of particular genes. The output of our analysis is bipartite. For each gene we provide

1. the parameters of the goodness of fit: log likelihood (log L), AIC and QE of the predicted mRNA levels with respect to the observed values

2. the corresponding regulators with their regulatory effect expressed by the local network weights; positive weights correspond to activator genes and negative weights correspond to repressor genes.

**Figure 1:**
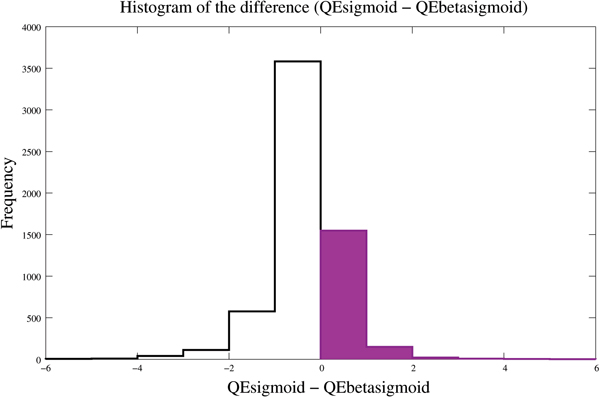
**Distribution of the difference between the quadratic errors of predictions**. The difference between the quadratic error of the sigmoid model and the quadratic error of the beta sigmoid error; the histogram on the positive part of the axis accounts for 29% from the total of genes.

**Figure 2:**
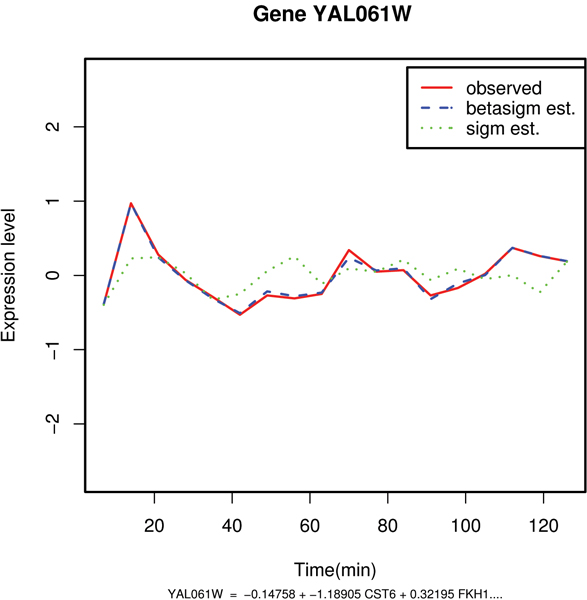
**Comparative plot between the observed and the predicted values of mRNA expression levels of gene YALO61W**. Example of good estimation of the expression profile with the beta sigmoid pattern of regulation: gene YAL061W.

**Figure 3:**
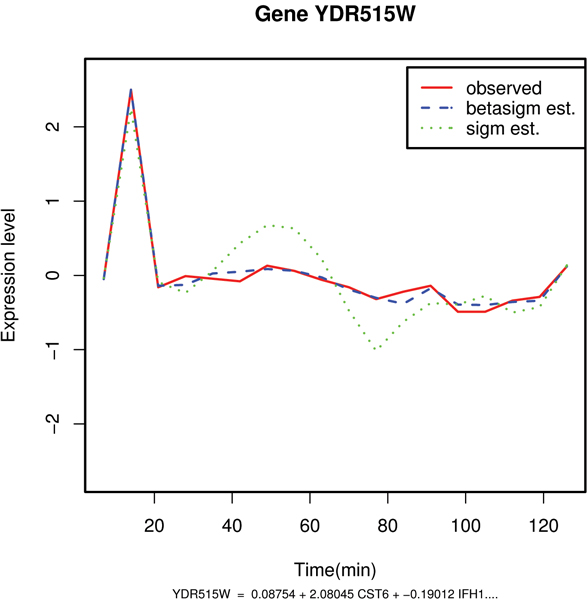
**Comparative plot between the observed and the predicted values of mRNA expression levels of gene YDR515W**. Example of good estimation of the expression profile with the beta sigmoid pattern of regulation: gene YDR515W.

**Figure 4:**
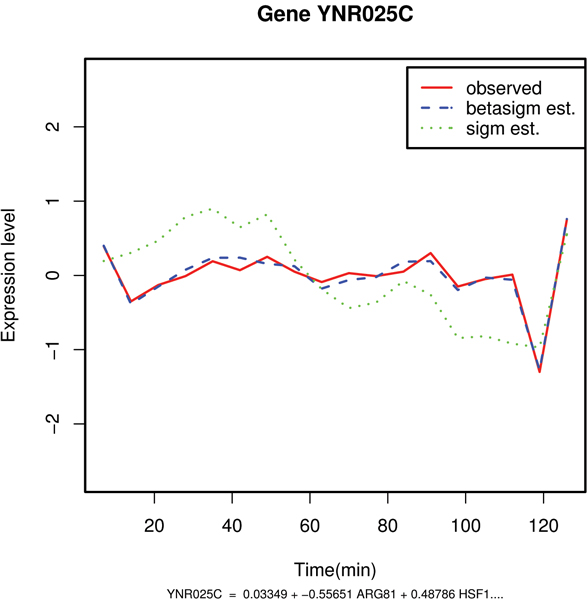
**Comparative plot between the observed and the predicted values of mRNA expression levels of gene YNR025C**. Example of good estimation of the expression profile with the beta sigmoid pattern of regulation: gene YNR025C.

The full output of our analysis is presented in Additional file 1. The beta sigmoid function was non-degenerate for 72% of the expression values. Tables 1 and 2 show that the use of the beta sigmoid model for the regulatory function improves the fitting parameters for 15 of the 20 genes depicted in [6] as worst fitted. The prediction of five genes (YGR269W, FIT3, HSH49, ASH1 and ATS1) shows a substantial amelioration. Over the entire data set, the novel model of regulatory function improved the prediction of 29% of the gene expression profiles. The distribution of the improvement is presented in the histogram in Figure 1 computed for the difference between the quadratic error of the predicted mRNA levels with the sigmoid model and the quadratic error of the predicted mRNA levels with the beta sigmoid model. We note that there is a non-negligible number of genes with their expression level better fitted by the beta sigmoid regulatory pattern. The prediction results for a selection of 10 genes in this category are shown in Table 3 and Table 4. Figure 2, Figure 3, and Figure 4 show comparative plots of the expression profiles for observed data and predictions from the stochastic differential equation model, with beta sigmoid and sigmoid prototypes of regulatory function. Two conclusions may be immediately drawn:

• the SDE model can provide very good predictions of mRNA expression levels;

• there exist genes for which the SDE model with beta sigmoid regulatory function gives a better prediction than the SDE model with sigmoid regulatory function.

A good quality of fitting of a particular gene allows the consideration of the regulators associated by the model for further investigation such as DNA-binding sites or promoter architectures. The quadratic error of prediction with beta sigmoid regulatory function is less than 0.5 for 1885 genes from the entire data set (see Additional file 1).

## Discussion and conclusions

The global view of the regulatory network is a cascade model, with genes regulating genes regulating other genes at their turn [11]. The SDE model [6] revisited here addresses the network local connections, *i.e. *the strict neighborhood of one target gene. The drift term of the stochastic differential equation is given by the regulation rate which quantifies the local network architecture by a linear combination of regulatory functions of regulating genes. The choice of the regulatory function pattern is a central aspect of the model, since the fitting of the gene expression profiles is very sensitive with respect to the drift term of the SDE. This model has the ability to extract from a given set of potential regulators those that fit the target gene expression profile.

The prototype of regulatory function introduced in [6] has a sigmoid pattern, built on the statistical characteristics of mRNA expression levels – see Equation (14). By keeping track of the temporal pattern of regulation, we show that the prediction of target gene expression profiles is improved for 29% of genes tested. We propose a prototype of regulatory function supported by a beta sigmoid model, built on temporal parameters extracted from the expression profiles of the regulators – see Equation (13). The SDE method relies on the assumption that the best fit of the target expression profiles is informative for the identification of the regulators and of their contribution. Thus, for the study of a specific set of target genes, our prototype of regulatory function may give more accurate results and provides a switch for the model proposed in [6]. Conceptually the beta sigmoid model has the advantage to correspond to the biological process of regulation: the temporal window of the peak defined by the shape of the beta sigmoid function reflects features of the regulation mechanism.

The regulation of gene expression in eukaryotes is a complex phenomenon and various particularities from one type of gene to another may occur. Hence the regulatory pattern may vary from gene to gene [4]. This fact is revealed in our result which shows that there are genes for which we can choose the best model between the beta sigmoid and the sigmoid pattern while for other genes neither of them fits the data. Before reaching this conclusion one has to be aware about the limitation induced from the selection of the set of potential regulators since incomplete information at this level may deteriorate the results.

Further research on more complex and explicit regulatory functions are foreseen from the availability of data sets and studies on various experimental condition for the budding yeast (sporulation [3], diauxic shift, heat and cold shock, treatment with DTT, pheromone and DNA-damaging agents [12]). In this framework a challenging task could be the study of the existence of possible relationships between the type of regulation pattern and the gene specificity.

This work provides a second implementation of the algorithm based on the SDE model, enlarged with a new type of regulatory pattern. The predictions from the algorithm may be improved with better strategies for the selection of the candidate pool of regulators. Moreover, the algorithm is a potential tool for the investigation of the interactions between the regulators of a target gene, modeled with a drift term defined by a non linear combination of regulatory functions.

This study shows that the SDE framework constitutes a reliable tool for the analysis of the transcriptional regulatory networks, provided it is completed with a validation of the identified regulators by a promoter analysis.

## Models and methods

### SDE model of time-continuous gene expression data

Let *T *denote a discrete set that corresponds to the time instants of the gene expression measurements. Consider two stochastic processes defined for a given target gene, (*N*_*t*_)_*t*∈*T *_and (*X*_*t*_)_*t*∈*T *_that model, respectively, the variation in time of the target gene amount of mRNA and the variation in time of the expression level of mRNA. Let ℛ
 MathType@MTEF@5@5@+=feaafiart1ev1aaatCvAUfKttLearuWrP9MDH5MBPbIqV92AaeXatLxBI9gBamrtHrhAL1wy0L2yHvtyaeHbnfgDOvwBHrxAJfwnaebbnrfifHhDYfgasaacH8akY=wiFfYdH8Gipec8Eeeu0xXdbba9frFj0=OqFfea0dXdd9vqai=hGuQ8kuc9pgc9s8qqaq=dirpe0xb9q8qiLsFr0=vr0=vr0dc8meaabaqaciaacaGaaeqabaWaaeGaeaaakeaaimaacqWFBeIuaaa@377D@ be the set of potential regulators for the target gene. Denote by *g*_*t *_the function that models the transcription rate of the target gene at time *t*

*gt *: P
 MathType@MTEF@5@5@+=feaafiart1ev1aaatCvAUfeBSjuyZL2yd9gzLbvyNv2CaerbwvMCKfMBHbqedmvETj2BSbWenfgDOvwBHrxAJfwnHbqeg0uy0HwzTfgDPnwy1aqee0evGueE0jxyaibaieIgFLIOYR2NHOxjYhrPYhrPYpI8F4rqqrFfpeea0xe9Lq=Jc9vqaqpepm0xbbG8FasPYRqj0=yi0lXdbba9pGe9qqFf0dXdHuk9fr=xfr=xfrpiWZqaaeaabiGaaiaacaqabeaadaqacqaaaOqaaiaabcfaaaa@4003@(ℛ
 MathType@MTEF@5@5@+=feaafiart1ev1aaatCvAUfKttLearuWrP9MDH5MBPbIqV92AaeXatLxBI9gBamrtHrhAL1wy0L2yHvtyaeHbnfgDOvwBHrxAJfwnaebbnrfifHhDYfgasaacH8akY=wiFfYdH8Gipec8Eeeu0xXdbba9frFj0=OqFfea0dXdd9vqai=hGuQ8kuc9pgc9s8qqaq=dirpe0xb9q8qiLsFr0=vr0=vr0dc8meaabaqaciaacaGaaeqabaWaaeGaeaaakeaaimaacqWFBeIuaaa@377D@) → ℛ
 MathType@MTEF@5@5@+=feaafiart1ev1aaatCvAUfKttLearuWrP9MDH5MBPbIqV92AaeXatLxBI9gBamrtHrhAL1wy0L2yHvtyaeHbnfgDOvwBHrxAJfwnaebbnrfifHhDYfgasaacH8akY=wiFfYdH8Gipec8Eeeu0xXdbba9frFj0=OqFfea0dXdd9vqai=hGuQ8kuc9pgc9s8qqaq=dirpe0xb9q8qiLsFr0=vr0=vr0dc8meaabaqaciaacaGaaeqabaWaaeGaeaaakeaaimaacqWFBeIuaaa@377D@_+ _    (1)

where P
 MathType@MTEF@5@5@+=feaafiart1ev1aaatCvAUfeBSjuyZL2yd9gzLbvyNv2CaerbwvMCKfMBHbqedmvETj2BSbWenfgDOvwBHrxAJfwnHbqeg0uy0HwzTfgDPnwy1aqee0evGueE0jxyaibaieIgFLIOYR2NHOxjYhrPYhrPYpI8F4rqqrFfpeea0xe9Lq=Jc9vqaqpepm0xbbG8FasPYRqj0=yi0lXdbba9pGe9qqFf0dXdHuk9fr=xfr=xfrpiWZqaaeaabiGaaiaacaqabeaadaqacqaaaOqaaiaabcfaaaa@4003@(ℛ
 MathType@MTEF@5@5@+=feaafiart1ev1aaatCvAUfKttLearuWrP9MDH5MBPbIqV92AaeXatLxBI9gBamrtHrhAL1wy0L2yHvtyaeHbnfgDOvwBHrxAJfwnaebbnrfifHhDYfgasaacH8akY=wiFfYdH8Gipec8Eeeu0xXdbba9frFj0=OqFfea0dXdd9vqai=hGuQ8kuc9pgc9s8qqaq=dirpe0xb9q8qiLsFr0=vr0=vr0dc8meaabaqaciaacaGaaeqabaWaaeGaeaaakeaaimaacqWFBeIuaaa@377D@) is the set of all possible subsets of ℛ
 MathType@MTEF@5@5@+=feaafiart1ev1aaatCvAUfKttLearuWrP9MDH5MBPbIqV92AaeXatLxBI9gBamrtHrhAL1wy0L2yHvtyaeHbnfgDOvwBHrxAJfwnaebbnrfifHhDYfgasaacH8akY=wiFfYdH8Gipec8Eeeu0xXdbba9frFj0=OqFfea0dXdd9vqai=hGuQ8kuc9pgc9s8qqaq=dirpe0xb9q8qiLsFr0=vr0=vr0dc8meaabaqaciaacaGaaeqabaWaaeGaeaaakeaaimaacqWFBeIuaaa@377D@ and ℛ
 MathType@MTEF@5@5@+=feaafiart1ev1aaatCvAUfKttLearuWrP9MDH5MBPbIqV92AaeXatLxBI9gBamrtHrhAL1wy0L2yHvtyaeHbnfgDOvwBHrxAJfwnaebbnrfifHhDYfgasaacH8akY=wiFfYdH8Gipec8Eeeu0xXdbba9frFj0=OqFfea0dXdd9vqai=hGuQ8kuc9pgc9s8qqaq=dirpe0xb9q8qiLsFr0=vr0=vr0dc8meaabaqaciaacaGaaeqabaWaaeGaeaaakeaaimaacqWFBeIuaaa@377D@_+ _is the set of real positive numbers. Denote the real, positive mRNA degradation rate by *λ*.

The model proposed in [6] assumes that from time *t *to Δ*t *the transcription and degradation process are given by

Nt+Δt−NtNt=(gt−λ)Δt+σΔWt     (2)
 MathType@MTEF@5@5@+=feaafiart1ev1aaatCvAUfKttLearuWrP9MDH5MBPbIqV92AaeXatLxBI9gBamXvP5wqSXMqHnxAJn0BKvguHDwzZbqegyvzYrwyUfgarqqtubsr4rNCHbGeaGqiA8vkIkVAFgIELiFeLkFeLk=iY=Hhbbf9v8qqaqFr0xc9pk0xbba9q8WqFfeaY=biLkVcLq=JHqVepeea0=as0db9vqpepesP0xe9Fve9Fve9GapdbaqaaeGacaGaaiaabeqaamqadiabaaGcbaWaaSaaaeaacqWGobGtdaWgaaWcbaGaemiDaqNaey4kaSIaeuiLdqKaemiDaqhabeaakiabgkHiTiabd6eaonaaBaaaleaacqWG0baDaeqaaaGcbaGaemOta40aaSbaaSqaaiabdsha0bqabaaaaOGaeyypa0JaeiikaGIaem4zaC2aaSbaaSqaaiabdsha0bqabaGccqGHsisliiGacqWF7oaBcqGGPaqkcqqHuoarcqWG0baDcqGHRaWkcqWFdpWCcqqHuoarcqWGxbWvdaWgaaWcbaGaemiDaqhabeaakiaaxMaacaWLjaWaaeWaaeaacqaIYaGmaiaawIcacaGLPaaaaaa@5FDD@

where (*W*_*t*_)_*t*∈*T *_is a Brownian Motion process that models the random error and *σ *is a positive scaling parameter. Consider infinitesimal time intervals, that is Δ*t *→ 0; from this it follows that the relation in Equation (2) becomes a stochastic differential equation

dNtNt=(gt−λ)dt+σdWt     (3)
 MathType@MTEF@5@5@+=feaafiart1ev1aaatCvAUfKttLearuWrP9MDH5MBPbIqV92AaeXatLxBI9gBaebbnrfifHhDYfgasaacH8akY=wiFfYdH8Gipec8Eeeu0xXdbba9frFj0=OqFfea0dXdd9vqai=hGuQ8kuc9pgc9s8qqaq=dirpe0xb9q8qiLsFr0=vr0=vr0dc8meaabaqaciaacaGaaeqabaqabeGadaaakeaadaWcaaqaaiabdsgaKjabd6eaonaaBaaaleaacqWG0baDaeqaaaGcbaGaemOta40aaSbaaSqaaiabdsha0bqabaaaaOGaeyypa0JaeiikaGIaem4zaC2aaSbaaSqaaiabdsha0bqabaGccqGHsisliiGacqWF7oaBcqGGPaqkcqWGKbazcqWG0baDcqGHRaWkcqWFdpWCcqWGKbazcqWGxbWvdaWgaaWcbaGaemiDaqhabeaakiaaxMaacaWLjaWaaeWaaeaacqaIZaWmaiaawIcacaGLPaaaaaa@4955@

Since *N*_*t *_is proportional with the signal intensity *S*_*t*_, and *X*_*t *_= log(*S*_*t *_- *B*) – where *B *is the background intensity – assume without loss of generality that

*X*_*t *_= log(*N*_*t*_)     (4)

Thus, the chain rule of the stochastic calculus applies (Itô formula) and the SDE obtained for *X*_*t *_yields

dXt=(gt−λ−σ22)dt+σdWt     (5)
 MathType@MTEF@5@5@+=feaafiart1ev1aaatCvAUfKttLearuWrP9MDH5MBPbIqV92AaeXatLxBI9gBaebbnrfifHhDYfgasaacH8akY=wiFfYdH8Gipec8Eeeu0xXdbba9frFj0=OqFfea0dXdd9vqai=hGuQ8kuc9pgc9s8qqaq=dirpe0xb9q8qiLsFr0=vr0=vr0dc8meaabaqaciaacaGaaeqabaqabeGadaaakeaacqWGKbazcqWGybawdaWgaaWcbaGaemiDaqhabeaakiabg2da9maabmaabaGaem4zaC2aaSbaaSqaaiabdsha0bqabaGccqGHsisliiGacqWF7oaBcqGHsisldaWcaaqaaiab=n8aZnaaCaaaleqabaGaeGOmaidaaaGcbaGaeGOmaidaaaGaayjkaiaawMcaaiabdsgaKjabdsha0jabgUcaRiab=n8aZjabdsgaKjabdEfaxnaaBaaaleaacqWG0baDaeqaaOGaaCzcaiaaxMaadaqadaqaaiabiwda1aGaayjkaiaawMcaaaaa@4B3E@

### Local regulatory network

Consider an increasing sequence of temporal values

*T *= {*t*_0 _<*t*_1 _<...<*t*_*n*_}     (6)

Let *m *be the cardinality of the set ℛ
 MathType@MTEF@5@5@+=feaafiart1ev1aaatCvAUfKttLearuWrP9MDH5MBPbIqV92AaeXatLxBI9gBamrtHrhAL1wy0L2yHvtyaeHbnfgDOvwBHrxAJfwnaebbnrfifHhDYfgasaacH8akY=wiFfYdH8Gipec8Eeeu0xXdbba9frFj0=OqFfea0dXdd9vqai=hGuQ8kuc9pgc9s8qqaq=dirpe0xb9q8qiLsFr0=vr0=vr0dc8meaabaqaciaacaGaaeqabaWaaeGaeaaakeaaimaacqWFBeIuaaa@377D@ and let Xti
 MathType@MTEF@5@5@+=feaafiart1ev1aaatCvAUfKttLearuWrP9MDH5MBPbIqV92AaeXatLxBI9gBaebbnrfifHhDYfgasaacH8akY=wiFfYdH8Gipec8Eeeu0xXdbba9frFj0=OqFfea0dXdd9vqai=hGuQ8kuc9pgc9s8qqaq=dirpe0xb9q8qiLsFr0=vr0=vr0dc8meaabaqaciaacaGaaeqabaqabeGadaaakeaacqWGybawdaqhaaWcbaGaemiDaqhabaGaemyAaKgaaaaa@30DE@ be the mRNA expression level of the *i*-th regulator from the set ℛ
 MathType@MTEF@5@5@+=feaafiart1ev1aaatCvAUfKttLearuWrP9MDH5MBPbIqV92AaeXatLxBI9gBamrtHrhAL1wy0L2yHvtyaeHbnfgDOvwBHrxAJfwnaebbnrfifHhDYfgasaacH8akY=wiFfYdH8Gipec8Eeeu0xXdbba9frFj0=OqFfea0dXdd9vqai=hGuQ8kuc9pgc9s8qqaq=dirpe0xb9q8qiLsFr0=vr0=vr0dc8meaabaqaciaacaGaaeqabaWaaeGaeaaakeaaimaacqWFBeIuaaa@377D@, measured at time *t *∈ *T*. Denote

X¯i=(Xt0i,Xt1i,...,Xtni)     (7)
 MathType@MTEF@5@5@+=feaafiart1ev1aaatCvAUfKttLearuWrP9MDH5MBPbIqV92AaeXatLxBI9gBaebbnrfifHhDYfgasaacH8akY=wiFfYdH8Gipec8Eeeu0xXdbba9frFj0=OqFfea0dXdd9vqai=hGuQ8kuc9pgc9s8qqaq=dirpe0xb9q8qiLsFr0=vr0=vr0dc8meaabaqaciaacaGaaeqabaqabeGadaaakeaadaadaaqaaiabdIfaybaadaahaaWcbeqaaiabdMgaPbaakiabg2da9iabcIcaOiabdIfaynaaDaaaleaacqWG0baDdaWgaaadbaGaeGimaadabeaaaSqaaiabdMgaPbaakiabcYcaSiabdIfaynaaDaaaleaacqWG0baDdaWgaaadbaGaeGymaedabeaaaSqaaiabdMgaPbaakiabcYcaSiabc6caUiabc6caUiabc6caUiabcYcaSiabdIfaynaaDaaaleaacqWG0baDdaWgaaadbaGaemOBa4gabeaaaSqaaiabdMgaPbaakiabcMcaPiaaxMaacaWLjaWaaeWaaeaacqaI3aWnaiaawIcacaGLPaaaaaa@4BF3@

**Figure 5:**
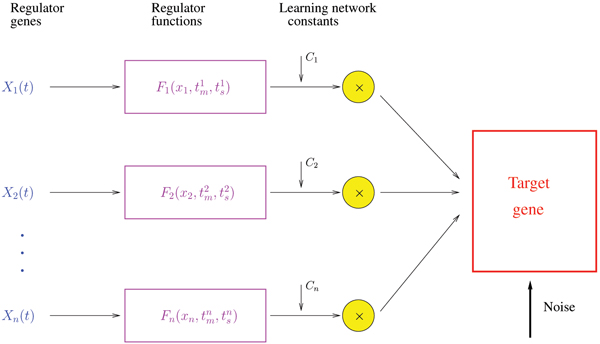
**Diagram of the local regulatory network model**. The model of the dependencies for the transcriptional regulatory network associated with a target gene.

The regulatory network is represented locally, in the neighborhood of the target gene, as a superposition of regulatory elements pictured in Figure 5. The local network relationship is modeled by the regulatory rate function, built from the observable information, i.e. the regulators mRNA expression levels, as:

gt=c0+∑i=1mciFi(X¯i,t)     (8)
 MathType@MTEF@5@5@+=feaafiart1ev1aaatCvAUfKttLearuWrP9MDH5MBPbIqV92AaeXatLxBI9gBaebbnrfifHhDYfgasaacH8akY=wiFfYdH8Gipec8Eeeu0xXdbba9frFj0=OqFfea0dXdd9vqai=hGuQ8kuc9pgc9s8qqaq=dirpe0xb9q8qiLsFr0=vr0=vr0dc8meaabaqaciaacaGaaeqabaqabeGadaaakeaacqWGNbWzdaWgaaWcbaGaemiDaqhabeaakiabg2da9iabdogaJnaaBaaaleaacqaIWaamaeqaaOGaey4kaSYaaabCaeaacqWGJbWydaWgaaWcbaGaemyAaKgabeaakiabdAeagnaaBaaaleaacqWGPbqAaeqaaOGaeiikaGYaaWaaaeaacqWGybawaaWaaWbaaSqabeaacqWGPbqAaaGccqGGSaalcqWG0baDcqGGPaqkaSqaaiabdMgaPjabg2da9iabigdaXaqaaiabd2gaTbqdcqGHris5aOGaaCzcaiaaxMaadaqadaqaaiabiIda4aGaayjkaiaawMcaaaaa@4B34@

where *F*_*i *_denotes the regulatory functions of the potential regulators from ℛ
 MathType@MTEF@5@5@+=feaafiart1ev1aaatCvAUfKttLearuWrP9MDH5MBPbIqV92AaeXatLxBI9gBamrtHrhAL1wy0L2yHvtyaeHbnfgDOvwBHrxAJfwnaebbnrfifHhDYfgasaacH8akY=wiFfYdH8Gipec8Eeeu0xXdbba9frFj0=OqFfea0dXdd9vqai=hGuQ8kuc9pgc9s8qqaq=dirpe0xb9q8qiLsFr0=vr0=vr0dc8meaabaqaciaacaGaaeqabaWaaeGaeaaakeaaimaacqWFBeIuaaa@377D@. The constants *c*_0_, *c*_1_,...,*c*_*m *_are the learning parameters of the network; they modulate the network behavior and carry information about the local regulatory process.

### Beta sigmoid pattern of regulation

The regulatory function is a key element of the model and fits the quantitative pattern with a specific regulator that acts on the mRNA expression of the target gene.

Our work revealed a prototype of the regulatory function based on the beta sigmoid function, given by

β(X¯i,t)=xmaxi[1+tmi−ttmi−tsi](ttmi)tmitmi−tsi,where     (9)
 MathType@MTEF@5@5@+=feaafiart1ev1aaatCvAUfKttLearuWrP9MDH5MBPbIqV92AaeXatLxBI9gBaebbnrfifHhDYfgasaacH8akY=wiFfYdH8Gipec8Eeeu0xXdbba9frFj0=OqFfea0dXdd9vqai=hGuQ8kuc9pgc9s8qqaq=dirpe0xb9q8qiLsFr0=vr0=vr0dc8meaabaqaciaacaGaaeqabaqabeGadaaakeaaiiGacqWFYoGycqGGOaakdaadaaqaaiabdIfaybaadaahaaWcbeqaaiabdMgaPbaakiabcYcaSiabdsha0jabcMcaPiabg2da9Gqabiab+Hha4naaDaaaleaacqGFTbqBcqGFHbqycqGF4baEaeaacqWGPbqAaaGcdaWadaqaaiabigdaXiabgUcaRmaalaaabaGae4hDaq3aa0baaSqaaiab+1gaTbqaaiabdMgaPbaakiabgkHiTiabdsha0bqaaiab+rha0naaDaaaleaacqGFTbqBaeaacqWGPbqAaaGccqGHsislcqGF0baDdaqhaaWcbaGae43CamhabaGaemyAaKgaaaaaaOGaay5waiaaw2faamaabmaabaWaaSaaaeaacqWG0baDaeaacqGF0baDdaqhaaWcbaGae4xBa0gabaGaemyAaKgaaaaaaOGaayjkaiaawMcaamaaCaaaleqabaWaaSaaaeaacqGF0baDdaqhaaadbaGae4xBa0gabaGaemyAaKgaaaWcbaGae4hDaq3aa0baaWqaaiab+1gaTbqaaiabdMgaPbaaliabgkHiTiab+rha0naaDaaameaacqGFZbWCaeaacqWGPbqAaaaaaaaakiabcYcaSiabbEha3jabbIgaOjabbwgaLjabbkhaYjabbwgaLjaaxMaacaWLjaWaaeWaaeaacqaI5aqoaiaawIcacaGLPaaaaaa@72DA@

xmaxi=max⁡{Xti|t∈T}     (10)
 MathType@MTEF@5@5@+=feaafiart1ev1aaatCvAUfKttLearuWrP9MDH5MBPbIqV92AaeXatLxBI9gBaebbnrfifHhDYfgasaacH8akY=wiFfYdH8Gipec8Eeeu0xXdbba9frFj0=OqFfea0dXdd9vqai=hGuQ8kuc9pgc9s8qqaq=dirpe0xb9q8qiLsFr0=vr0=vr0dc8meaabaqaciaacaGaaeqabaqabeGadaaakeaaieqacqWF4baEdaqhaaWcbaGae8xBa0Mae8xyaeMae8hEaGhabaGaemyAaKgaaOGaeyypa0JagiyBa0MaeiyyaeMaeiiEaG3aaiWaaeaacqWGybawdaqhaaWcbaGaemiDaqhabaGaemyAaKgaaOGaeiiFaWNaemiDaqNaeyicI4SaemivaqfacaGL7bGaayzFaaGaaCzcaiaaxMaadaqadaqaaiabigdaXiabicdaWaGaayjkaiaawMcaaaaa@49C2@

tmi=min⁡{t∈T|Xti=xmaxi}     (11)
 MathType@MTEF@5@5@+=feaafiart1ev1aaatCvAUfKttLearuWrP9MDH5MBPbIqV92AaeXatLxBI9gBaebbnrfifHhDYfgasaacH8akY=wiFfYdH8Gipec8Eeeu0xXdbba9frFj0=OqFfea0dXdd9vqai=hGuQ8kuc9pgc9s8qqaq=dirpe0xb9q8qiLsFr0=vr0=vr0dc8meaabaqaciaacaGaaeqabaqabeGadaaakeaaieqacqWF0baDdaqhaaWcbaGae8xBa0gabaGaemyAaKgaaOGaeyypa0JagiyBa0MaeiyAaKMaeiOBa42aaiWaaeaacqWG0baDcqGHiiIZcqWGubavcqGG8baFcqWGybawdaqhaaWcbaGaemiDaqhabaGaemyAaKgaaOGaeyypa0Jae8hEaG3aa0baaSqaaiab=1gaTjab=fgaHjab=Hha4bqaaiabdMgaPbaaaOGaay5Eaiaaw2haaiaaxMaacaWLjaWaaeWaaeaacqaIXaqmcqaIXaqmaiaawIcacaGLPaaaaaa@4F24@

tsi=arg⁡max⁡{ΔXti|t∈T}     (12)
 MathType@MTEF@5@5@+=feaafiart1ev1aaatCvAUfKttLearuWrP9MDH5MBPbIqV92AaeXatLxBI9gBaebbnrfifHhDYfgasaacH8akY=wiFfYdH8Gipec8Eeeu0xXdbba9frFj0=OqFfea0dXdd9vqai=hGuQ8kuc9pgc9s8qqaq=dirpe0xb9q8qiLsFr0=vr0=vr0dc8meaabaqaciaacaGaaeqabaqabeGadaaakeaaieqacqWF0baDdaqhaaWcbaGae83CamhabaGaemyAaKgaaOGaeyypa0JagiyyaeMaeiOCaiNaei4zaCMagiyBa0MaeiyyaeMaeiiEaG3aaiWaaeaacqqHuoarcqWGybawdaqhaaWcbaGaemiDaqhabaGaemyAaKgaaOGaeiiFaWNaemiDaqNaeyicI4SaemivaqfacaGL7bGaayzFaaGaaCzcaiaaxMaadaqadaqaaiabigdaXiabikdaYaGaayjkaiaawMcaaaaa@4C82@

**Figure 6:**
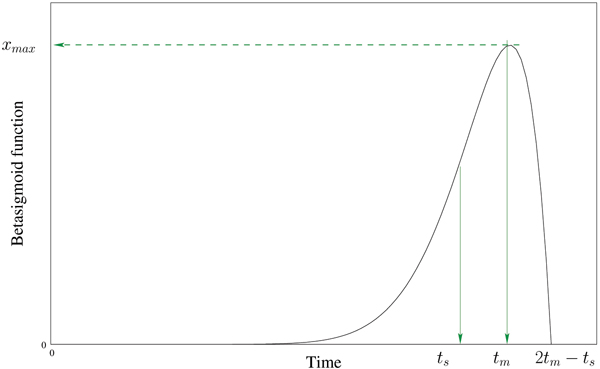
**Beta sigmoid function shape**. Example of beta sigmoid function with narrow support.

Figure 6 shows an example of beta sigmoid function shape. The parameter tsi
 MathType@MTEF@5@5@+=feaafiart1ev1aaatCvAUfKttLearuWrP9MDH5MBPbIqV92AaeXatLxBI9gBaebbnrfifHhDYfgasaacH8akY=wiFfYdH8Gipec8Eeeu0xXdbba9frFj0=OqFfea0dXdd9vqai=hGuQ8kuc9pgc9s8qqaq=dirpe0xb9q8qiLsFr0=vr0=vr0dc8meaabaqaciaacaGaaeqabaqabeGadaaakeaaieqacqWF0baDdaqhaaWcbaGae83CamhabaGaemyAaKgaaaaa@3116@ corresponds to the point where the regulator expression level begins to increase. The maximal contribution of the regulator *i *is induced in the target gene at time tmi
 MathType@MTEF@5@5@+=feaafiart1ev1aaatCvAUfKttLearuWrP9MDH5MBPbIqV92AaeXatLxBI9gBaebbnrfifHhDYfgasaacH8akY=wiFfYdH8Gipec8Eeeu0xXdbba9frFj0=OqFfea0dXdd9vqai=hGuQ8kuc9pgc9s8qqaq=dirpe0xb9q8qiLsFr0=vr0=vr0dc8meaabaqaciaacaGaaeqabaqabeGadaaakeaaieqacqWF0baDdaqhaaWcbaGae8xBa0gabaGaemyAaKgaaaaa@310A@, when the mRNA expression level of the regulator attends its maximum, corresponding to the biological hypotheses. The beta sigmoid function degenerates after time 2tmi
 MathType@MTEF@5@5@+=feaafiart1ev1aaatCvAUfKttLearuWrP9MDH5MBPbIqV92AaeXatLxBI9gBaebbnrfifHhDYfgasaacH8akY=wiFfYdH8Gipec8Eeeu0xXdbba9frFj0=OqFfea0dXdd9vqai=hGuQ8kuc9pgc9s8qqaq=dirpe0xb9q8qiLsFr0=vr0=vr0dc8meaabaqaciaacaGaaeqabaqabeGadaaakeaaieqacqWF0baDdaqhaaWcbaGae8xBa0gabaGaemyAaKgaaaaa@310A@ - tsi
 MathType@MTEF@5@5@+=feaafiart1ev1aaatCvAUfKttLearuWrP9MDH5MBPbIqV92AaeXatLxBI9gBaebbnrfifHhDYfgasaacH8akY=wiFfYdH8Gipec8Eeeu0xXdbba9frFj0=OqFfea0dXdd9vqai=hGuQ8kuc9pgc9s8qqaq=dirpe0xb9q8qiLsFr0=vr0=vr0dc8meaabaqaciaacaGaaeqabaqabeGadaaakeaaieqacqWF0baDdaqhaaWcbaGae83CamhabaGaemyAaKgaaaaa@3116@ and becomes non-informative. For this reason we define the regulatory function as

Fi(X¯i,t)=I{β(X¯i,t)>0}(X¯i,t)β(X¯i,t)+I{β(X¯i,t)≤0}(X¯i,t)η(X¯i,t)     (13)
 MathType@MTEF@5@5@+=feaafiart1ev1aaatCvAUfKttLearuWrP9MDH5MBPbIqV92AaeXatLxBI9gBaebbnrfifHhDYfgasaacH8akY=wiFfYdH8Gipec8Eeeu0xXdbba9frFj0=OqFfea0dXdd9vqai=hGuQ8kuc9pgc9s8qqaq=dirpe0xb9q8qiLsFr0=vr0=vr0dc8meaabaqaciaacaGaaeqabaqabeGadaaakeaacqWGgbGrdaWgaaWcbaGaemyAaKgabeaakiabcIcaOmaamaaabaGaemiwaGfaamaaCaaaleqabaGaemyAaKgaaOGaeiilaWIaemiDaqNaeiykaKIaeyypa0dcbeGae8xsaK0aaSbaaSqaaiabcUha7HGaciab+j7aIjabcIcaOmaamaaabaGaemiwaGfaamaaCaaameqabaGaemyAaKgaaSGaeiilaWIaemiDaqNaeiykaKIaeyOpa4JaeGimaaJaeiyFa0habeaakiabcIcaOmaamaaabaGaemiwaGfaamaaCaaaleqabaGaemyAaKgaaOGaeiilaWIaemiDaqNaeiykaKIae4NSdiMaeiikaGYaaWaaaeaacqWGybawaaWaaWbaaSqabeaacqWGPbqAaaGccqGGSaalcqWG0baDcqGGPaqkcqGHRaWkcqWFjbqsdaWgaaWcbaGaei4EaSNae4NSdiMaeiikaGYaaWaaaeaacqWGybawaaWaaWbaaWqabeaacqWGPbqAaaWccqGGSaalcqWG0baDcqGGPaqkcqGHKjYOcqaIWaamcqGG9bqFaeqaaOGaeiikaGYaaWaaaeaacqWGybawaaWaaWbaaSqabeaacqWGPbqAaaGccqGGSaalcqWG0baDcqGGPaqkcqGF3oaAcqGGOaakdaadaaqaaiabdIfaybaadaahaaWcbeqaaiabdMgaPbaakiabcYcaSiabdsha0jabcMcaPiaaxMaacaWLjaWaaeWaaeaacqaIXaqmcqaIZaWmaiaawIcacaGLPaaaaaa@79C1@

where **I**_*A *_is the indicator function of the set A (**I**_*A*_(*x*) = 1 if *x *∈ *A *and **I**_*A*_(*x*) = 0 if *x *∉ *A*) and

η(X¯i,t)=11+e−(Xti−μi)/σi     (14)
 MathType@MTEF@5@5@+=feaafiart1ev1aaatCvAUfKttLearuWrP9MDH5MBPbIqV92AaeXatLxBI9gBaebbnrfifHhDYfgasaacH8akY=wiFfYdH8Gipec8Eeeu0xXdbba9frFj0=OqFfea0dXdd9vqai=hGuQ8kuc9pgc9s8qqaq=dirpe0xb9q8qiLsFr0=vr0=vr0dc8meaabaqaciaacaGaaeqabaqabeGadaaakeaaiiGacqWF3oaAcqGGOaakdaadaaqaaiabdIfaybaadaahaaWcbeqaaiabdMgaPbaakiabcYcaSiabdsha0jabcMcaPiabg2da9maalaaabaGaeGymaedabaGaeGymaeJaey4kaSIaemyzau2aaWbaaSqabeaacqGHsislcqGGOaakcqWGybawdaqhaaadbaGaemiDaqhabaGaemyAaKgaaSGaeyOeI0Iae8hVd02aaSbaaWqaaiabdMgaPbqabaWccqGGPaqkcqGGVaWlcqWFdpWCdaWgaaadbaGaemyAaKgabeaaaaaaaOGaaCzcaiaaxMaadaqadaqaaiabigdaXiabisda0aGaayjkaiaawMcaaaaa@4E8C@

is the sigmoid function; *μ*_*i *_and *σ*_*i *_are the mean and deviation of X¯i
 MathType@MTEF@5@5@+=feaafiart1ev1aaatCvAUfKttLearuWrP9MDH5MBPbIqV92AaeXatLxBI9gBaebbnrfifHhDYfgasaacH8akY=wiFfYdH8Gipec8Eeeu0xXdbba9frFj0=OqFfea0dXdd9vqai=hGuQ8kuc9pgc9s8qqaq=dirpe0xb9q8qiLsFr0=vr0=vr0dc8meaabaqaciaacaGaaeqabaqabeGadaaakeaadaadaaqaaiabdIfaybaadaahaaWcbeqaaiabdMgaPbaaaaa@2F7D@, the prototype of the regulatory function from [6].

The learning in the local network is driven by the SDE

dXt=[c˜0+∑i=1nciFi(Xti)]dt+σdWt     (15)
 MathType@MTEF@5@5@+=feaafiart1ev1aaatCvAUfKttLearuWrP9MDH5MBPbIqV92AaeXatLxBI9gBaebbnrfifHhDYfgasaacH8akY=wiFfYdH8Gipec8Eeeu0xXdbba9frFj0=OqFfea0dXdd9vqai=hGuQ8kuc9pgc9s8qqaq=dirpe0xb9q8qiLsFr0=vr0=vr0dc8meaabaqaciaacaGaaeqabaqabeGadaaakeaacqWGKbazcqWGybawdaWgaaWcbaGaemiDaqhabeaakiabg2da9maadmaabaGafm4yamMbaGaadaWgaaWcbaGaeGimaadabeaakiabgUcaRmaaqahabaGaem4yam2aaSbaaSqaaiabdMgaPbqabaGccqWGgbGrdaWgaaWcbaGaemyAaKgabeaakiabcIcaOiabdIfaynaaDaaaleaacqWG0baDaeaacqWGPbqAaaGccqGGPaqkaSqaaiabdMgaPjabg2da9iabigdaXaqaaiabd6gaUbqdcqGHris5aaGccaGLBbGaayzxaaGaemizaqMaemiDaqNaey4kaSccciGae83WdmNaemizaqMaem4vaC1aaSbaaSqaaiabdsha0bqabaGccaWLjaGaaCzcamaabmaabaGaeGymaeJaeGynaudacaGLOaGaayzkaaaaaa@5801@

where c˜
 MathType@MTEF@5@5@+=feaafiart1ev1aaatCvAUfKttLearuWrP9MDH5MBPbIqV92AaeXatLxBI9gBaebbnrfifHhDYfgasaacH8akY=wiFfYdH8Gipec8Eeeu0xXdbba9frFj0=OqFfea0dXdd9vqai=hGuQ8kuc9pgc9s8qqaq=dirpe0xb9q8qiLsFr0=vr0=vr0dc8meaabaqaciaacaGaaeqabaqabeGadaaakeaacuWGJbWygaacaaaa@2E0A@_0 _= *c*_0 _- *λ *- *σ*^2^/2. The network weights *c*_1_,...,*c*_*m *_carry information in both their magnitude and sign: positive values correspond to regulators with activation, and negative values correspond to repression.

### Statistical analysis

For a given target gene, the aim of the statistical analysis is to extract from the time course mRNA levels

1. the set of *m *regulators (model selection);

2. their corresponding parameters *σ *and the set {c˜
 MathType@MTEF@5@5@+=feaafiart1ev1aaatCvAUfKttLearuWrP9MDH5MBPbIqV92AaeXatLxBI9gBaebbnrfifHhDYfgasaacH8akY=wiFfYdH8Gipec8Eeeu0xXdbba9frFj0=OqFfea0dXdd9vqai=hGuQ8kuc9pgc9s8qqaq=dirpe0xb9q8qiLsFr0=vr0=vr0dc8meaabaqaciaacaGaaeqabaqabeGadaaakeaacuWGJbWygaacaaaa@2E0A@_0_, *c*_1_,...,*c*_*m*_} of parameters estimation;

with the best fit with respect to Equation (15). The beta sigmoid as regulatory function adds supplementary parameters tsi
 MathType@MTEF@5@5@+=feaafiart1ev1aaatCvAUfKttLearuWrP9MDH5MBPbIqV92AaeXatLxBI9gBaebbnrfifHhDYfgasaacH8akY=wiFfYdH8Gipec8Eeeu0xXdbba9frFj0=OqFfea0dXdd9vqai=hGuQ8kuc9pgc9s8qqaq=dirpe0xb9q8qiLsFr0=vr0=vr0dc8meaabaqaciaacaGaaeqabaqabeGadaaakeaaieqacqWF0baDdaqhaaWcbaGae83CamhabaGaemyAaKgaaaaa@3116@, tmi
 MathType@MTEF@5@5@+=feaafiart1ev1aaatCvAUfKttLearuWrP9MDH5MBPbIqV92AaeXatLxBI9gBaebbnrfifHhDYfgasaacH8akY=wiFfYdH8Gipec8Eeeu0xXdbba9frFj0=OqFfea0dXdd9vqai=hGuQ8kuc9pgc9s8qqaq=dirpe0xb9q8qiLsFr0=vr0=vr0dc8meaabaqaciaacaGaaeqabaqabeGadaaakeaaieqacqWF0baDdaqhaaWcbaGae8xBa0gabaGaemyAaKgaaaaa@310A@ and **x**_**max **_to the model. These parameters are estimated from the corresponding time course mRNA levels according to their definitions given in Equation (10) and employed in the computation of the estimators of *σ *and c¯
 MathType@MTEF@5@5@+=feaafiart1ev1aaatCvAUfKttLearuWrP9MDH5MBPbIqV92AaeXatLxBI9gBaebbnrfifHhDYfgasaacH8akY=wiFfYdH8Gipec8Eeeu0xXdbba9frFj0=OqFfea0dXdd9vqai=hGuQ8kuc9pgc9s8qqaq=dirpe0xb9q8qiLsFr0=vr0=vr0dc8meaabaqaciaacaGaaeqabaqabeGadaaakeaacuWGJbWygaqhaaaa@2E1F@:

For the evaluation of the impact of the beta sigmoid regulatory function model, the network weights are estimated from gene expression data with the standard statistical procedure described in detail in [6]. Equation (15) is considered in discrete form for each time interval [*t*_*j*_, *t*_*j*+1_], *j *= {1, 2,...,*n*} that corresponds to time measurements. The estimators of *σ *and {c˜
 MathType@MTEF@5@5@+=feaafiart1ev1aaatCvAUfKttLearuWrP9MDH5MBPbIqV92AaeXatLxBI9gBaebbnrfifHhDYfgasaacH8akY=wiFfYdH8Gipec8Eeeu0xXdbba9frFj0=OqFfea0dXdd9vqai=hGuQ8kuc9pgc9s8qqaq=dirpe0xb9q8qiLsFr0=vr0=vr0dc8meaabaqaciaacaGaaeqabaqabeGadaaakeaacuWGJbWygaacaaaa@2E0A@_0_, *c*_1_,...,*c*_*m*_} are obtained maximizing the log-likelihood function log *L *(ML approach [13]) of the *n*-dimensional random vector with elements

Xtj+1−Xtjtj+1−tj.
 MathType@MTEF@5@5@+=feaafiart1ev1aaatCvAUfKttLearuWrP9MDH5MBPbIqV92AaeXatLxBI9gBaebbnrfifHhDYfgasaacH8akY=wiFfYdH8Gipec8Eeeu0xXdbba9frFj0=OqFfea0dXdd9vqai=hGuQ8kuc9pgc9s8qqaq=dirpe0xb9q8qiLsFr0=vr0=vr0dc8meaabaqaciaacaGaaeqabaqabeGadaaakeaadaWcaaqaaiabdIfaynaaBaaaleaacqWG0baDdaWgaaadbaGaemOAaOMaey4kaSIaeGymaedabeaaaSqabaGccqGHsislcqWGybawdaWgaaWcbaGaemiDaq3aaSbaaWqaaiabdQgaQbqabaaaleqaaaGcbaWaaOaaaeaacqWG0baDdaWgaaWcbaGaemOAaOMaey4kaSIaeGymaedabeaakiabgkHiTiabdsha0naaBaaaleaacqWGQbGAaeqaaaqabaaaaOGaeiOla4caaa@4220@

The computation of log *L *uses basic properties of Brownian Motion: the increments Wtj+1−Wtj
 MathType@MTEF@5@5@+=feaafiart1ev1aaatCvAUfKttLearuWrP9MDH5MBPbIqV92AaeXatLxBI9gBaebbnrfifHhDYfgasaacH8akY=wiFfYdH8Gipec8Eeeu0xXdbba9frFj0=OqFfea0dXdd9vqai=hGuQ8kuc9pgc9s8qqaq=dirpe0xb9q8qiLsFr0=vr0=vr0dc8meaabaqaciaacaGaaeqabaqabeGadaaakeaacqWGxbWvdaWgaaWcbaGaemiDaq3aaSbaaWqaaiabdQgaQjabgUcaRiabigdaXaqabaaaleqaaOGaeyOeI0Iaem4vaC1aaSbaaSqaaiabdsha0naaBaaameaacqWGQbGAaeqaaaWcbeaaaaa@3847@ are pairwise independent and each increment is normally distributed, with zero mean and standard deviation given by tj+1−tj
 MathType@MTEF@5@5@+=feaafiart1ev1aaatCvAUfKttLearuWrP9MDH5MBPbIqV92AaeXatLxBI9gBaebbnrfifHhDYfgasaacH8akY=wiFfYdH8Gipec8Eeeu0xXdbba9frFj0=OqFfea0dXdd9vqai=hGuQ8kuc9pgc9s8qqaq=dirpe0xb9q8qiLsFr0=vr0=vr0dc8meaabaqaciaacaGaaeqabaqabeGadaaakeaadaGcaaqaaiabdsha0naaBaaaleaacqWGQbGAcqGHRaWkcqaIXaqmaeqaaOGaeyOeI0IaemiDaq3aaSbaaSqaaiabdQgaQbqabaaabeaaaaa@3579@.

The criteria used for the selection of the regulators is AIC [14]. Between any two combinations of regulators, the best combination is that for which the AIC of the regulators has the smallest value. The computation of AIC follows from

*AIC *= -2log⁡L^
 MathType@MTEF@5@5@+=feaafiart1ev1aaatCvAUfKttLearuWrP9MDH5MBPbIqV92AaeXatLxBI9gBaebbnrfifHhDYfgasaacH8akY=wiFfYdH8Gipec8Eeeu0xXdbba9frFj0=OqFfea0dXdd9vqai=hGuQ8kuc9pgc9s8qqaq=dirpe0xb9q8qiLsFr0=vr0=vr0dc8meaabaqaciaacaGaaeqabaqabeGadaaakeaadaqiaaqaaiGbcYgaSjabc+gaVjabcEgaNjabdYeambGaayPadaaaaa@32AD@ + 2(*m *+ 1)

where log⁡L^
 MathType@MTEF@5@5@+=feaafiart1ev1aaatCvAUfKttLearuWrP9MDH5MBPbIqV92AaeXatLxBI9gBaebbnrfifHhDYfgasaacH8akY=wiFfYdH8Gipec8Eeeu0xXdbba9frFj0=OqFfea0dXdd9vqai=hGuQ8kuc9pgc9s8qqaq=dirpe0xb9q8qiLsFr0=vr0=vr0dc8meaabaqaciaacaGaaeqabaqabeGadaaakeaadaqiaaqaaiGbcYgaSjabc+gaVjabcEgaNjabdYeambGaayPadaaaaa@32AD@ is the estimator of log *L *and is obtained from the functional invariance property of the maximum likelihood estimators σ^
 MathType@MTEF@5@5@+=feaafiart1ev1aaatCvAUfKttLearuWrP9MDH5MBPbIqV92AaeXatLxBI9gBaebbnrfifHhDYfgasaacH8akY=wiFfYdH8Gipec8Eeeu0xXdbba9frFj0=OqFfea0dXdd9vqai=hGuQ8kuc9pgc9s8qqaq=dirpe0xb9q8qiLsFr0=vr0=vr0dc8meaabaqaciaacaGaaeqabaqabeGadaaakeaaiiGacuWFdpWCgaqcaaaa@2E86@ and c¯^
 MathType@MTEF@5@5@+=feaafiart1ev1aaatCvAUfKttLearuWrP9MDH5MBPbIqV92AaeXatLxBI9gBaebbnrfifHhDYfgasaacH8akY=wiFfYdH8Gipec8Eeeu0xXdbba9frFj0=OqFfea0dXdd9vqai=hGuQ8kuc9pgc9s8qqaq=dirpe0xb9q8qiLsFr0=vr0=vr0dc8meaabaqaciaacaGaaeqabaqabeGadaaakeaacuWGJbWygaqhgaqcaaaa@2E2E@, i.e.,

log⁡L^
 MathType@MTEF@5@5@+=feaafiart1ev1aaatCvAUfKttLearuWrP9MDH5MBPbIqV92AaeXatLxBI9gBaebbnrfifHhDYfgasaacH8akY=wiFfYdH8Gipec8Eeeu0xXdbba9frFj0=OqFfea0dXdd9vqai=hGuQ8kuc9pgc9s8qqaq=dirpe0xb9q8qiLsFr0=vr0=vr0dc8meaabaqaciaacaGaaeqabaqabeGadaaakeaadaqiaaqaaiGbcYgaSjabc+gaVjabcEgaNjabdYeambGaayPadaaaaa@32AD@ = log *L*(σ^
 MathType@MTEF@5@5@+=feaafiart1ev1aaatCvAUfKttLearuWrP9MDH5MBPbIqV92AaeXatLxBI9gBaebbnrfifHhDYfgasaacH8akY=wiFfYdH8Gipec8Eeeu0xXdbba9frFj0=OqFfea0dXdd9vqai=hGuQ8kuc9pgc9s8qqaq=dirpe0xb9q8qiLsFr0=vr0=vr0dc8meaabaqaciaacaGaaeqabaqabeGadaaakeaaiiGacuWFdpWCgaqcaaaa@2E86@, c¯^
 MathType@MTEF@5@5@+=feaafiart1ev1aaatCvAUfKttLearuWrP9MDH5MBPbIqV92AaeXatLxBI9gBaebbnrfifHhDYfgasaacH8akY=wiFfYdH8Gipec8Eeeu0xXdbba9frFj0=OqFfea0dXdd9vqai=hGuQ8kuc9pgc9s8qqaq=dirpe0xb9q8qiLsFr0=vr0=vr0dc8meaabaqaciaacaGaaeqabaqabeGadaaakeaacuWGJbWygaqhgaqcaaaa@2E2E@).

Let ℋ
 MathType@MTEF@5@5@+=feaafiart1ev1aaatCvAUfKttLearuWrP9MDH5MBPbIqV92AaeXatLxBI9gBaebbnrfifHhDYfgasaacH8akY=wiFfYdH8Gipec8Eeeu0xXdbba9frFj0=OqFfea0dXdd9vqai=hGuQ8kuc9pgc9s8qqaq=dirpe0xb9q8qiLsFr0=vr0=vr0dc8meaabaqaciaacaGaaeqabaqabeGadaaakeaat0uy0HwzTfgDPnwy1egaryqtHrhAL1wy0L2yHvdaiqaacqWFlecsaaa@3762@ denote the set formed by a candidate pool of regulators of the target gene; denote by |ℋ
 MathType@MTEF@5@5@+=feaafiart1ev1aaatCvAUfKttLearuWrP9MDH5MBPbIqV92AaeXatLxBI9gBaebbnrfifHhDYfgasaacH8akY=wiFfYdH8Gipec8Eeeu0xXdbba9frFj0=OqFfea0dXdd9vqai=hGuQ8kuc9pgc9s8qqaq=dirpe0xb9q8qiLsFr0=vr0=vr0dc8meaabaqaciaacaGaaeqabaqabeGadaaakeaat0uy0HwzTfgDPnwy1egaryqtHrhAL1wy0L2yHvdaiqaacqWFlecsaaa@3762@| the cardinality of ℋ
 MathType@MTEF@5@5@+=feaafiart1ev1aaatCvAUfKttLearuWrP9MDH5MBPbIqV92AaeXatLxBI9gBaebbnrfifHhDYfgasaacH8akY=wiFfYdH8Gipec8Eeeu0xXdbba9frFj0=OqFfea0dXdd9vqai=hGuQ8kuc9pgc9s8qqaq=dirpe0xb9q8qiLsFr0=vr0=vr0dc8meaabaqaciaacaGaaeqabaqabeGadaaakeaat0uy0HwzTfgDPnwy1egaryqtHrhAL1wy0L2yHvdaiqaacqWFlecsaaa@3762@. Ideally, ML and AIC procedures shall be performed on each combination of regulators from ℋ
 MathType@MTEF@5@5@+=feaafiart1ev1aaatCvAUfKttLearuWrP9MDH5MBPbIqV92AaeXatLxBI9gBaebbnrfifHhDYfgasaacH8akY=wiFfYdH8Gipec8Eeeu0xXdbba9frFj0=OqFfea0dXdd9vqai=hGuQ8kuc9pgc9s8qqaq=dirpe0xb9q8qiLsFr0=vr0=vr0dc8meaabaqaciaacaGaaeqabaqabeGadaaakeaat0uy0HwzTfgDPnwy1egaryqtHrhAL1wy0L2yHvdaiqaacqWFlecsaaa@3762@. Since the number of all possible combinations of regulators is 2|ℋ|
 MathType@MTEF@5@5@+=feaafiart1ev1aaatCvAUfKttLearuWrP9MDH5MBPbIqV92AaeXatLxBI9gBaebbnrfifHhDYfgasaacH8akY=wiFfYdH8Gipec8Eeeu0xXdbba9frFj0=OqFfea0dXdd9vqai=hGuQ8kuc9pgc9s8qqaq=dirpe0xb9q8qiLsFr0=vr0=vr0dc8meaabaqaciaacaGaaeqabaqabeGadaaakeaacqaIYaGmdaahaaWcbeqaaiabcYha8nrtHrhAL1wy0L2yHvtyaeHbnfgDOvwBHrxAJfwnaGabaiab=TqiijabcYha8baaaaa@3B81@, an enumeration algorithm for those sets will explode quickly. The heuristic procedure used is the forward selection strategy [15]. At first the regulator with the biggest log-likelihood with respect to the target gene is selected. A new regulator is added if it will increase the AIC more than any other single regulator outside the current combination. The actual implementation stops for a combination of maximum 10 regulators, exactly as done in [6]. Under these conditions the performance of the algorithm we propose is expressed by an order of magnitude equal to *O*(*nm*^2^). In practice this is a slight enhancement compared to the algorithm proposed in [6] for which the order of magnitude equals *O*(*n*^2^*m*^2^) – since for actual experimental data the number of time courses *n *is quite small. The difference in the performance of the two algorithms comes from the fact that the search of the maximum is less costly than the computation of the statistical parameters for a data set.

## List of abbreviations used

SDE: Stochastic Differential Equation

AIC: Akaike Information Criteria

ML: Maximum Likelihood

QE: Quadratic Error

## Availability

The method was implemented in R 2.2.1 (R Development Core Team, ). The source code is available upon request.

## Competing interests

The authors declare that they have no competing interests.

## Authors' contributions

ACH devised the model, performed statistical analysis and drafted the manuscript. MDQ implemented the model, collected the data and edited the manuscript. Both authors read and approved the final manuscript.

## Supplementary Material

Additional File 1 Lists of strings (for the gene names) and numerical data (for their fitting parameters). The full table obtained from processing with the SDE beta sigmoid method the Spellman data [1] Click here for file

## Figures and Tables

**Table 1:** List of genes reported as worst fitted in [6] and their prediction results from the SDE beta sigmoid model

Target	logL	AIC	QE	Best Fit
YBR089W(NA)	-3.13	10.27	16.56	YBR089W = -0.180 + 0.481 BAS1
YDR285W(ZIP1)*	1.25	5.48	2.17	YDR285W = 0.253 + -0.258 GAT1 + -0.511 GCN4 + -0.202 FKH1
YFR057W(NA)*	2.33	1.32	2.40	YFR057W = -0.057 + 0.405 GAL80 + -0.09129 IFH1
YAL018C(NA)*	5.29	-0.58	1.25	YAL018C = 0.256 + -1.985 GAL80 + -0.922 FKH2 + 1.983 IME1 + 0.624 HMS1
YOR264W(DSE3)*	4.65	-3.31	2.77	YOR264W = -0.265 + 0.205 CHA4 + 0.684 IME1
YOL116W(MSN1)*	3.29	-0.58	3.58	YOL116W = -0.045 + 0.229 HAL9 + -0.132 FZF1
YGR269W(NA)**	12.41	-14.83	0.48	YGR269W = 0.011 + -0.263 GZF3 + 0.313 CRZ1 + -0.383 DAL80 + 0.361 AZF1
YOR383C(FIT3)**	10.07	-9.73	0.81	YOR383C = 0.073 + -0.199 ARG81+ 0.325 GLN3 + -0.218IFH1
YOR319W(HSH49)**	11.52	-11.05	0.60	YOR319W = -0.033 + 0.337 CST6 + -0.170 CIN5 + 0.719 GAL80 + -0.191 IFH1 + -0.274 ACA1
YKL001C(MET14)*	2.26	-0.52	2.16	YKL001C = 0.05 + -0.141 MAC1
YDL117W(CYK3)*	8.73	-7.47	1.21	YDL117W = -0.463 + 0.577 AFT2 + 0.703 FKH2 + 0.177 HAP5 + 0.132 FAP7
YKL185W(ASH1)**	10.01	-10.02	0.52	YKL185W = 0.176 + -0.485 ASK10 + -0.241 DOT6 + 0.211 FAP7 + 0.163 HAP5
YBR158W(AMN1)	-0.53	9.06	6.40	YBR158W = 0.066 + -0.191 IFH1 + 0.392 CHA4 + -0.313 ABF1
YBR108W(NA)*	9.23	-8.47	0.98	YBR108W = 0.082 + 0.723 HMS1 + -1.512 GAL80 + 1.58 IME1 + -0.639 FKH2
YAL020C(ATS1)**	9.84	-11.69	0.61	YAL020C = 0.011 + -0.267 HAC1 + 0.534 GAL4 + -0.521 INO4
YBR002C(RER2)	3.41	-0.83	3.46	YBR002C = -0.020 + 0.239 FKH1 + -0.229 ABF1
YCL040W(GLK1)*	6.78	-7.56	3.13	YCL040W = -0.118 + 0.261 CST6 + 0.300 HMS1
YNL018C(NA)*	5.15	-4.31	1.43	YNL018C = 0.028 + -0.259 KRE33 + 0.182 CAD1
YNL192W(CHS1)	1.64	2.71	3.45	YNL192W = -0.081 + 0.307 CHA4 + -0.182 ARG81
YBR230C(NA)	0.74	6.51	2.20	YBR230C = -0.025 + 0.625 MAC1 + -0.694 HAP2 + 0.462 HOG1

Fitting parameters obtained with the beta sigmoid model for the set of the 20 worst fitted genes with the sigmoid regulatory model; the asterisk * marks an improvement of the fitting with respect to the results from Table 2. The prediction of five genes (YGR269W, FIT3, HSH49, ASH1 and ATS1) shows a significant improvement.

**Table 2:** List of genes reported as worst fitted in [6] and their prediction results from the SDE sigmoid model

Target	logL	AIC	QE	Best Fit
YBR089W(NA)	-1.68	7.36	3.2	YBR089W = -0.166 + 0.367 HAA1
YDR285W(ZIP1)	0.77	2.46	3.69	YDR285W = 0.191 + -0.368 INO2
YFR057W(NA)	1.13	1.74	4.31	YFR057W = 0.098 + -0.188 GCN4
YAL018C(NA)	1.52	2.96	1.79	YAL018C = 0.055 + -0.303 IME1 + 0.195 CRZ1
YOR264W(DSE3)	2.26	-0.52	5.56	YOR264W = -0.059 + 0.129 ARG80
YOL116W(MSN1)	2.3	-0.59	3.77	YOL116W = -0.092 + 0.193 HAL9
YGR269W(NA)	2.4	-0.81	5.19	YGR269W = 0.097 + -0.194 HMS1
YOR383C(FIT3)	1.82	6.37	2.64	YOR383C = 0.367 + -0.287 ARG81 + -0.464 ECM22 + 0.412 GLN3 + -0.335 MAC1
YOR319W(HSH49)	2.17	5.65	4.92	YOR319W = 0.83 + -1.13 CIN5 + -0.655 FHL1 + 0.354 DAL81 + -0.275 FKH1
YKL001C(MET14)	2.58	-1.16	4.34	YKL001C = 0.091 + -0.18 IME1
YDL117W(CYK3)	2.59	-1.18	4.35	YDL117W = -0.162 + 0.359 AFT2
YKL185W(ASH1)	2.64	2.73	2.37	YKL185W = -0.150 + 0.407 ACE2 + -0.421 GAT1 + 0.302 INO2
YBR158W(AMN1)	2.65	8.7	1.2	YBR158W = -0.139 + 0.926 KRE33 + -0.941 IME4 + 0.571 MAL13 + 0.264 GAT3 + -0.347 CBF1 + -0.285 AZF1
YBR108W(NA)	2.66	-1.33	2.85	YBR108W = 0.112 + -0.205 HAC1
YAL020C(ATS1)	2.75	-1.51	4.15	YAL020C = -0.133 + 0.256 ASK10
YBR002C(RER2)	3.07	-2.14	2.26	YBR002C = 0.101 + -0.2 HAP5
YCL040W(GLK1)	3.09	-2.18	3.18	YCL040W = 0.095 + -0.199 HAL9
YNL018C(NA)	3.59	-3.18	2.19	YNL018C = 0.078 + -0.154 ARG81
YNL192W(CHS1)	3.21	1.57	2.13	YNL192W = -0.115 + 0.115 FZF1 + 0.306 DAL81 + -0.209 HMS2
YBR230C(NA)	3.32	3.37	2.2	YBR230C = -0.52 + 0.484 MAC1 + 0.467 GZF3 + 0.374 INO4 + -0.244 EDS1

The set of the worst fitted 20 genes by the sigmoid model, sorted in the increasing order of the log-likelihood.

**Table 3:** Prediction results from the SDE beta sigmoid model for selected genes

Target	logL	AIC	QE	Best Fit
YMR096W(SNZ1)	8.86	-11.72	0.8	YMR096W = -0.069 + 0.330 HAP3 + 0.115 CIN5
YNR025C(NA)	13.7	-13.4	0.11	YNR025C = 0.033 + -0.556 ARG81 + 0.487 HSF1+ 0.195 FAP7 + -0.120 FKH1 + -0.319 DAL81 + 0.141 GCR2
YPR200C(ARR2)	13.04	-14.08	0.29	YPR200C = 0.00037 + -0.707 GAL4 + 0.369 INO4 + 0.364 HAP2 + -0.201 ABF1 + 0.129 FAP7
YGR234W(YHB1)	15.27	-24.53	0.46	YGR234W = -0.042 + -0.157 HIR1 + 0.139 ABF1
YGR269W(NA)	12.42	-14.84	0.48	YGR269W = 0.011 + -0.263 GZF3 + 0.313 CRZ1 + -0.383 DAL80 + 0.361 AZF1
YGL150C(INO80)	16.71	-21.43	0.21	YGL150C = -0.237 + 0.197 CST6 + 0.368 GAT3 + 0.169 KRE33 + 0.185 ABF1 + -0.122 CAD1
YDR193W(NA)	10.67	-13.35	0.48	YDR193W = 0.044 + 0.731 CST6 + -0.141 IFH1 + -0.185 DOT6
YAL061W(NA)	21.24	-28.47	0.02	YAL061W = -0.147 + -1.189 CST6 + 0.321 FKH1 + -.369 IXR1+1.521 BYE1+.125 GAT3 +.165 ACA1
YKL150W(MCR1)	12.29	-16.57	0.41	YKL150W = 0.048 + 0.515 ACA1 + -0.222 HIR1 + -0.205 GAL80
YDR515W(SLF1)	19.88	-29.76	0.09	YDR515W = 0.087 + 2.080 CST6 + -0.190 IFH1 + -2.660 GTS1 + 0.956 FHL1

Genes fitted by the SDE model with beta sigmoid as regulatory function.

**Table 4:** Prediction results from the SDE sigmoid model corresponding to genes from Table 3

Target	logL	AIC	QE	Best Fit
YMR096W(SNZ1)	7.27	-8.54	6.16	YMR096W = -0.159 + 0.179 GCN4 + 0.174 HAA1
YNR025C(NA)	3.8	-1.61	5.42	YNR025C = 0.008 + -0.261 HMS1 + 0.278 ACA1
YPR200C(ARR2)	3.97	-3.94	5.28	YPR200C = 0.144 + -0.315 INO4
YGR234W(YHB1)	11.08	-18.17	5.28	YGR234W = 0.059 + -0.12 ARG81
YGR269W(NA)	2.4	-0.81	5.19	YGR269W = 0.097 + -0.194 HMS1
YGL150C(INO80)	4.25	-4.51	4.41	YGL150C = -0.082 + 0.168 GAT3
YDR193W(NA)	6.22	-0.44	4.45	YDR193W = -0.278 + 0.415 LEU3 + 0.166 GAL4 + -0.691 FAP7 + 0.293 CUP9 + 0.375 DAT1
YAL061W(NA)	8.07	-12.13	1.66	YAL061W = -0.087 + 0.191 CUP9
YKL150W(MCR1)	7.93	-9.86	3.84	YKL150W = 0.249 + -0.325 CBF1 + -0.175 HAA1
YDR515W(SLF1)	5.94	0.12	2	YDR515W = 0.246 + -0.562 CIN5 + -0.347 CBF1 + 0.256 HIR1 + 0.453 HAP4 + -0.304 IFH1

The fitting parameters from the sigmoid model of regulatory functions of the genes from Table 3.
